# Biotransformation of lanthanum by *Aspergillus niger*

**DOI:** 10.1007/s00253-018-9489-0

**Published:** 2018-11-15

**Authors:** Xia Kang, Laszlo Csetenyi, Geoffrey Michael Gadd

**Affiliations:** 1 Geomicrobiology Group, School of Life Sciences, University of Dundee, Dundee, Scotland DD1 5EH UK; 2 Concrete Technology Group, Department of Civil Engineering, University of Dundee, Dundee, Scotland DD1 4HN UK

**Keywords:** Rare earth elements, Lanthanum, *Aspergillus niger*, Biotransformation, Biorecovery

## Abstract

Lanthanum is an important rare earth element and has many applications in modern electronics and catalyst manufacturing. However, there exist several obstacles in the recovery and cycling of this element due to a low average grade in exploitable deposits and low recovery rates by energy-intensive extraction procedures. In this work, a novel method to transform and recover La has been proposed using the geoactive properties of *Aspergillus niger*. La-containing crystals were formed and collected after *A. niger* was grown on Czapek-Dox agar medium amended with LaCl_3_. Energy-dispersive X-ray analysis (EDXA) showed the crystals contained C, O, and La; scanning electron microscopy revealed that the crystals were of a tabular structure with terraced surfaces. X-ray diffraction identified the mineral phase of the sample as La_2_(C_2_O_4_)_3_·10H_2_O. Thermogravimetric analysis transformed the oxalate crystals into La_2_O_3_ with the kinetics of thermal decomposition corresponding well with theoretical calculations. Geochemical modelling further confirmed that the crystals were lanthanum decahydrate and identified optimal conditions for their precipitation. To quantify crystal production, biomass-free fungal culture supernatants were used to precipitate La. The results showed that the precipitated lanthanum decahydrate achieved optimal yields when the concentration of La was above 15 mM and that 100% La was removed from the system at 5 mM La. Our findings provide a new aspect in the biotransformation and biorecovery of rare earth elements from solution using biomass-free fungal culture systems.

## Introduction

Rare earth elements (REE) are vital to the world’s fastest growing markets including renewable energy, electric vehicles, telecommunication and defence technologies and the demand for them has risen year on year (Goodenough et al. 2018). Lanthanum is moderately abundant in the Earth’s crust and represents a group of REE that include 14 lanthanides that have multiple industrial applications such as additives in the production of high-performance alloys and as catalysts in glass and ceramics manufacturing and in the refinement of crude oil (Kilbourn 1987; Tyler 2004; Massari and Ruberti 2013). Lanthanum oxide (La_2_O_3_) is an important material in microelectronics as it exhibits the best electrical properties in metal oxide semiconductor field effect transistors and could be the ideal substitute for SiO_2_ in CMOS integrated circuits (Leskelä and Ritala 2003; Kakushima et al. 2007). Another potential application in environmental technology is that it can be used as a highly efficient absorbent for the removal of phosphorus from polluted water (Zhang et al. 2011; Xie et al. 2015). REE primarily occur in associated minerals that are scattered among other mineral-containing ores, meaning that mining can be costly and ineffective due to a lack of economically exploitable deposits worldwide, with the average grade of REE-containing ores being usually less than 10% (Humphries 2010). Traditional physico-chemical methods involve multiple steps of leaching with a combination of hazardous chemicals, e.g. nitric acid and butyl phosphonate, with a huge amount of energy consumption only achieving a recovery rate of approximately 1% (Preston et al. 1996). This is very low when compared with other industrially important elements such as platinum group metals (PGMs) where average recovery rates can amount to 96% or even higher (Patel and Dawson 2015). Therefore, new approaches for the recovery of REE from mines, leachates and waste materials need to be proposed, which could, if adopted for commercial use, greatly alleviate the shortage of supplies of these metals.

Through the mechanism of biologically induced mineralization (BIM) (Gadd 2010), whereby organisms create favourable conditions for extracellular chemical precipitation of mineral phases by modifying their extracellular microenvironment, metal-containing compounds can be formed. *Aspergillus niger* is a common soil fungus and an important industrial microorganism for the production of citric acid (Currie 1917; Kubicek and Rohr 1985; Mattey 1992; Papagianni 2007). It is also noted for producing various other organic acids including gluconic and oxalic acids, and all these can have a huge role in geomicrobial transformations of minerals and metal speciation (Gadd 1999; Gadd et al. 2014). The application of oxalic acid is an effective means by which certain metals can be precipitated in the form of insoluble metal oxalates of general formula M_*x*_(C_2_O_4_)_*y*_·zH_2_O. Bioprecipitation of metals mediated by oxalic acid-producing fungi, such as *A. niger*, can provide a potentially useful mechanism for metal biorecovery from solution (Ilyas et al. 2017; Liang and Gadd 2017). The recovery of elements is an indispensable step in the bioleaching process for both REE-bearing ores and waste materials (Ilyas et al. 2017). Some approaches to recovering REE from La-containing ores and electronic wastes by involving microbial participation have also been reported, e.g. bioleaching, bioaccumulation and biosorption (Ismail et al. 2015; Ayora et al. 2016; Park et al. 2016). Some previous studies have shown that REE can be mobilized from solid materials such as spent catalysts and luminescent powder from cathode ray tubes by sulphur-oxidizing bacteria, such as *Acidithiobacillus ferrooxidans*, *A. thiooxidans* and *Leptospirillum ferrooxidans* (Barmettler et al. 2016). In a separate study, 50% of the total REE was released from spent fluid catalytic cracking catalyst by 4-day-old cell-free supernatant of *Gluconobacter oxydans*, indicating that organic acid-producing microorganisms can effectively induce bioleaching and improve the recovery of REE from waste materials (Reed et al. 2016). Hopfe et al. (2017) discovered that significant amounts of REE were leached from lanthanide-containing fluorescent phosphor material using both a biomass-free supernatant and living culture of a mixture of yeasts and acetic acid bacteria. Some preliminary research has reported the dissolution of commercial lanthanide oxides (Schwartz and Näveke 1980), by different strains of *A. niger*, which confirmed the accessibility of lanthanides to *Aspergillus* species. Supernatants of cultures of *A. niger*, *A. ficuum* and *A. terreus* have been used by some researchers to study bioleaching of REE from natural sources, such as carbonaceous shales, monazite sand, red mud and Th–U concentrate (Hassanien et al. 2013; Amin et al. 2014; Qu et al. 2015; Brisson et al. 2016; Desouky et al. 2016; Keekan and Jalondhara 2017). REE in ores can be recovered as oxides through several steps including acid leaching, filtration, precipitation using oxalic acid and calcination, and this has been in practice as a part of the recovery process for some minerals such as bastnasite and monazite (Sinha et al. 2016). Despite fungi exhibiting a significant role in biogeochemical cycles for metals and metalloids (Gadd 2007), few studies have clarified interactions of fungal species with REE. Therefore, more information on the mechanisms and conditions effecting the biotransformation of REE is clearly needed. The present study examines the bioprecipitation of lanthanum oxalate by *A. niger* and adds new knowledge about the microbial biotransformation of lanthanides.

## Materials and methods

### Microorganism and media

The fungal strain used in this study was *Aspergillus niger* (ATCC 1015), which was routinely maintained in Petri dishes containing commercial malt extract agar (MEA) (Lab M Limited, Bury, Lancashire, UK) at 25 °C in the dark. Modified Czapek-Dox medium (MCD) contained the following substances (l^−1^ Milli-Q H_2_O): glucose 30 g, NaNO_3_ 3 g, Na_2_HPO_4_ 1 g, MgSO_4_·7H_2_O 0.5 g, KCl 0.5 g and FeSO_4_·7H_2_O 0.01 g. Where required, 15 g of agar no. 1 (Lab M, Bury, UK) was added to make solid agar medium. The final pH of MCD medium was adjusted to pH 5.5 using 1 M HCl/NaOH prior to autoclaving for 15 min at 115 °C. To achieve higher oxalate precipitation yields, nitrate (NO_3_^−^) was used as the nitrogen source in modified AP1 medium, composed of the following (l^−1^ Milli-Q H_2_O): NaNO_3_ 3 g, KH_2_PO_4_ 0.5 g, MgSO_4_·7H_2_O 0.2 g, CaCl_2_·6H_2_O 0.05 g, NaCl 0.1 g, FeCl_3_·6H_2_O 0.0025 g, trace metals (ZnSO_4_·7H_2_O 0.004 g, MnSO_4_·4H_2_O 0.004 g and CuSO_4_·5H_2_O 0.0004 g) and glucose 20 g. The medium was adjusted to pH 5.5 using 1 M HCl/NaOH prior to sterilization at 115 °C for 15 min. For preparation of solid plates, 15 g of agar no. 1 was applied to 1000 ml of medium.

### Lanthanum biotransformation

Lanthanum chloride heptahydrate (99.9%) (Sigma-Aldrich, St Louis, MO, USA) was used as the La source in experiments using solid agar plates. A 500-mM LaCl_3_ stock solution was prepared and sterilized through a Minisart Syringe Filter (pore size 0.2 μm) (Sartorius Lab Instruments GmbH & Co., Goettingen, Germany) before use. Each Petri dish contained 25-ml La-spiked medium, which was prepared by pipetting appropriate amounts of the La stock solution into the nutrient agar when cooled to 55 °C after autoclaving at 115 °C. The surface pH of the plates was measured using a flat-tipped pH probe (VWR International, Lutterworth, England, UK). A 90-mm-diameter cellophane membrane (Focus Packaging and Design Ltd., Louth, UK), which was treated by washing with Milli-Q water and autoclaving at least three times in Milli-Q water at 121 °C for 15 min, was placed on top of the agar surface to separate the fungal biomass from the medium. Plugs (6 mm diameter) were cut from the leading edge of a vigorously growing *A. niger* colony using a sterile cork borer and transferred to La-containing plates and incubated in the dark at 25 °C until the colony reached the edge of the plate. Colony diameter was measured daily in two directions across the colony. To recover the biomass, the fungal colony growing on the membrane was removed from the surface, washed three times with Milli-Q water and oven-dried at 105 °C until constant weight. Biomass yield was determined by measuring the dry weight; the surface pH after fungal growth was measured as described previously. A tolerance index (TI) was calculated according to the following formula: TI = (dry weight of La-exposed biomass/dry weight of control biomass × 100%) (Sayer et al. 1995; Wei et al. 2013). Growth was presented as colony expansion rates, growth rates being defined as millimetres per day, calculated according to a linear regression equation. Each treatment was conducted at least in triplicate.

Crystals that were formed in the agar were recovered by gently homogenizing the agar in 50 ml Milli-Q water at 80 °C in a crystallizing dish (Sayer and Gadd 1997; Li et al. 2015). After sedimentation and washing at least three times with cool Milli-Q water, the extracted crystals were stored in a desiccator until constant weight, and accurately measured using an HR-150A analytical balance (A&D Instruments Ltd., Abingdon, Oxfordshire, UK).

For lanthanum biotransformation experiments in liquid medium, *A. niger* was inoculated on MEA slants and grown for a few days until profuse growth resulted. To obtain a spore suspension, 20 ml of sterile 0.1% (*v*/*v*_aq_) Tween 80 was added to the slants and mixed well by vortexing. The resulting suspension was then filtered through sterile muslin cloth to remove mycelia from the suspension. The spores were washed three times using sterile Milli-Q water to remove remaining Tween 80 by centrifugation at 2553×*g* for 30 min. Serial dilution was carried out to obtain a spore concentration of 1 × 10^8^ ml^−1^; 1 ml of this inoculum was transferred to 99 ml liquid medium in a 250-ml Erlenmeyer flask, which was maintained in a shaking incubator (Infors Multitron Standard, Rittergasse, Switzerland) at 125 rpm in the dark at 25 °C for 7 days. For harvesting, the culture was aseptically filtered through sterile 63-μm nylon mesh (John Staniar & Co. Ltd., Manchester, UK) to obtain a biomass-free supernatant which was stored at 4 °C prior to experiments. Supernatant pH was measured as described previously.

La biotransformations by fungal supernatants were performed in 15 ml centrifuge tubes, incubated on a roller mixer (Stuart Equipment, Stone, Staffordshire, UK) at room temperature for 24 h, prepared by adding 1 ml sterile LaCl_3_ of the desired concentration into 9 ml fungal supernatant to achieve a final volume of 10 ml and La concentrations of 0, 1, 3, 5, 7, 11, 15, 20, 30, 40 and 50 mM. After precipitation, minerals produced were collected by sedimentation and removal of the supernatant and then washed three times with Milli-Q water before being dried in a desiccator at room temperature until constant weight, in order to calculate crystal yield. Each treatment was performed at least in triplicate.

### Crystal yield and La assay

To accurately estimate precipitation yield and removal of La from the supernatant after precipitation, dried crystal samples were accurately weighed as previously described. After fungal supernatant reactions with LaCl_3_, resulting supernatants were collected by centrifugation (2553×*g*, 30 min) prior to measurement of lanthanum concentrations using the Arsenazo III colorimetric method (White and Gadd 1990; Rohwer et al. 1995; Ivanov and Ermakova 2001) and an Ultrospec 2100 pro spectrophotometer (Biochrom Ltd., Holliston, MA, USA) at an optical density of 658 nm. This was achieved by pipetting 1-ml sample into a test tube with 1 ml 0.02% (*w*/*v*) Arsenazo III (Sigma-Aldrich, St. Louis, USA) in pH 2.8 potassium hydrogen phthalate buffer with a final volume of 10 ml made up by adding Milli-Q water. The concentration of lanthanum in the solution was calculated based on a calibration curve created using a series of standard solutions at 0, 1.44, 3.60, 7.20, 10.80 and 14.40 μmol l^−1^ La. The La removal rate was defined as R_La_ = (*m*_1_ − *m*_2_) × 100%, where *m*_1_ is the amount of La added to the reaction system and *m*_2_ that of the remaining La in the supernatant after reaction.

### Geochemical modelling

Geochemical modelling was carried out to analyse interactions of lanthanum with oxalate and pH in the aqueous state using Geochemist’s Workbench (GWB) edition 12.0 (Aqueous Solutions LLC, Urbana-Champaign, USA), which is mainly comprised of two subprograms, i.e. SpecE8 for calculating species activities and Act2 for creating stability diagrams (Ceci et al. 2015a, b; Li and Gadd 2017). The stability diagrams of both (C_2_O_4_)_2_^−^ and La^3+^ activities as a function of pH were constructed using Act2 to simulate changes in the mineral species under ideal conditions; all ion activities were calculated using SpecE8 according to their concentrations in the liquid medium. As *A. niger* was found to secrete ~ 25 mM oxalic acid after 12-day growth in a previous study (Ceci et al. 2015b), the activity of oxalate was calculated based on 25 mM (C_2_O_4_)_2_^−^ in the equilibrium system, while that of lanthanum was set at 1 mM La^3+^, which was found to be an effective concentration to precipitate La as oxalate. Activities for other chemicals in simulated spent AP1 medium were calculated using the following concentrations: 35.3 mM NaNO_3_, 3.7 mM KH_2_PO_4_, 1.7 mM NaCl, 0.8 mM MgSO_4_·7H_2_O, 0.2 mM CaCl_2_·6H_2_O, 17.9 μM MnSO_4_·4H_2_O, 13.9 μM ZnSO_4_·7H_2_O, 9.3 μM FeCl_3_·6H_2_O and 1.6 μM CuSO_4_·5H_2_O. For MCD fungal supernatant, the concentrations used were the following: 35.3 mM NaNO_3_, 7.0 mM Na_2_HPO_4_, 6.7 mM KCl, 2.0 mM MgSO_4_·7H_2_O and 36.0 μM FeSO_4_·7H_2_O. All simulated equilibrium conditions were set at 25 °C under 1.013 bars atmospheric pressure.

### EDXA and SEM

Dried La-containing crystals were mounted on adhesive carbon tape stuck to aluminium electron microscopy specimen stubs (JEOL, 25 mm × 8 mm) for determination of elemental composition using an energy-dispersive X-ray spectroscopy system (Oxford Instruments Inca, Abingdon, Oxfordshire, UK) embedded within a JEOL JSM 7400F field emission scanning electron microscope (JEOL Ltd., Tokyo, Japan) operating at an accelerating voltage of 15 kV for 100 s. Samples on the stub were plated with a layer of 10-nm gold and platinum using a Cressington 208HR sputter coater (Cressington Scientific Instruments Ltd., Watford, England, UK) prior to examination using the emission scanning electron microscope (JEOL JSM7400F) running at an accelerating voltage of 5 kV.

### XRD and TGA

Crystal samples were ground to a fine powder using a clean ceramic mortar and pestle and compacted tightly on the reverse side of an aluminium specimen holder (15 × 20 × 2 mm^3^), which was held against a glass side and detached after the back cover was snapped into place. The mineral phase of the crystals was identified using a Hiltonbrooks X-ray diffractometer (HiltonBrooks Ltd., Crewe, UK) equipped with a single graphite crystal monochromatic CuKα chronometer (30 mA, 40 kV). Duplicate samples were analysed over the range of 3–60° 2*θ* angle at a scan rate of one degree per min in 0.1° increments.

TGA of the crystal samples was carried out using a NETZSCH STA 409PC TG/DTG/DTA analyser fitted with a SiC furnace (NETZSCH Group, Selb, Germany). Small amounts (< 100 mg) of crystals were heated to 1000 °C at a heating rate of 10 K min^−1^ using dry N_2_ as a purge gas at a flow rate of 100 cm^3^ min^−1^. The samples were maintained at 1000 °C until constant weight. A curve of mass loss as a function of temperature was created as the result. After thermal treatment, all samples were collected for XRD analysis to identify the presence of possible mineral phases.

### Statistical analysis

One-way ANOVA was applied using Tukey’s test to compare means of growth rate, biomass yield, and surface pH at a significance level of 0.05 between samples from the same medium treated with different concentrations of lanthanum.

## Results

### Fungal growth and crystal formation

**Fig. 1:**
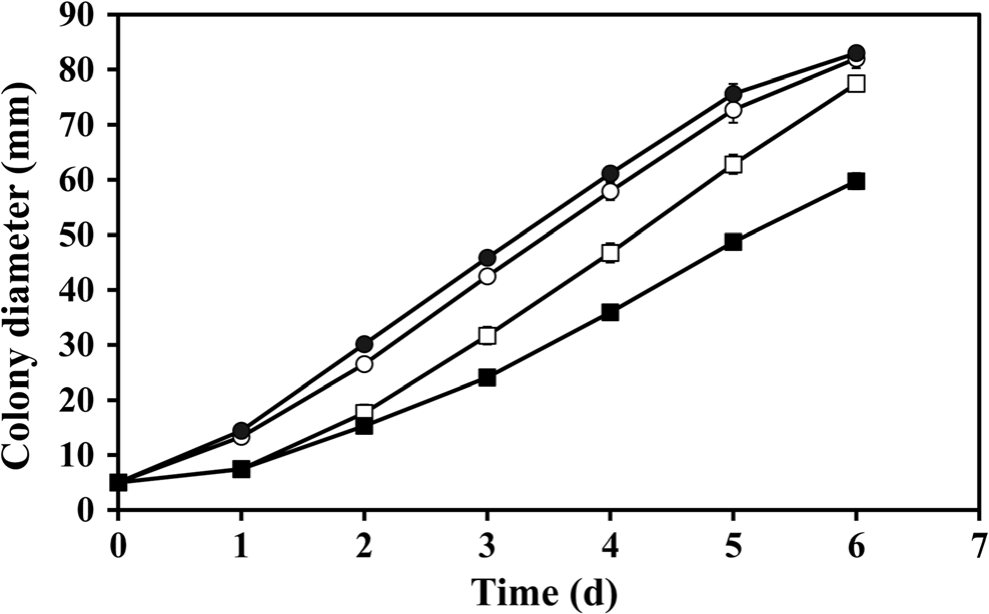
Colony expansion rate of *A. niger* on solid Czapek-Dox media containing 0 mM (empty circle), 1 mM (filled circle), 5 mM (empty square) and 10 mM (filled square) LaCl_3_ over 6-day incubation at 25 °C in the dark. Data are averages of at least three replicates with error bars (shown only when greater than symbol dimensions) showing the standard error of the mean

Measurement of colony diameters was discontinued on the 6th day of growth when the *A. niger* colonies reached the edge of the plate. *A. niger* grew on La-free MCD plates at an average rate of 14.21 ± 0.46 mm day^−1^ which was not significantly influenced (*p* < 0.05) by the presence of 1 and 5 mM LaCl_3_. However, at 10 mM LaCl_3_, the growth rate was reduced approximately by 25% compared with the control. Biomass yield decreased slightly from 0.19 ± 0.01 to 0.17 ± 0.01 g dry wt as the La concentration increased (Fig. 1). The agar surface pH was pH 1.86 ± 0.04 for the La-free control and pH 1.71 ± 0.04 for the 10 mM La plates. The most prominent decrease in the tolerance index occurred at 5 mM La, where the biomass yield was lowered by 12.4% (Table 1).

**Table 1:** Growth rate, biomass yield, agar surface pH and tolerance index (TI) of *A. niger* after 14-day growth at 25 °C in the dark in solid Czapek-Dox medium supplemented with lanthanum chloride

La concentration (mM)	Growth rate (mm day^−1^)	Biomass (g dry wt)	Surface pH	TI (%)
0	14.21 ± 0.46a	0.19 ± 0.01a	1.86 ± 0.04b	100
1	14.12 ± 0.11a	0.18 ± 0.05a	2.00 ± 0.04a	99.0
5	14.31 ± 0.21a	0.16 ± 0.01a	1.87 ± 0.03b	87.6
10	10.67 ± 0.33b	0.17 ± 0.01a	1.71 ± 0.04c	92.1

Tolerance index (TI) was calculated according to the following formula: TI = (dry weight of La-exposed biomass/dry weight of control biomass × 100%). Data are given as means ± SD from three independent replicates; different lowercase letters in each column indicate the significance level at *p* < 0.05

**Fig. 2:**
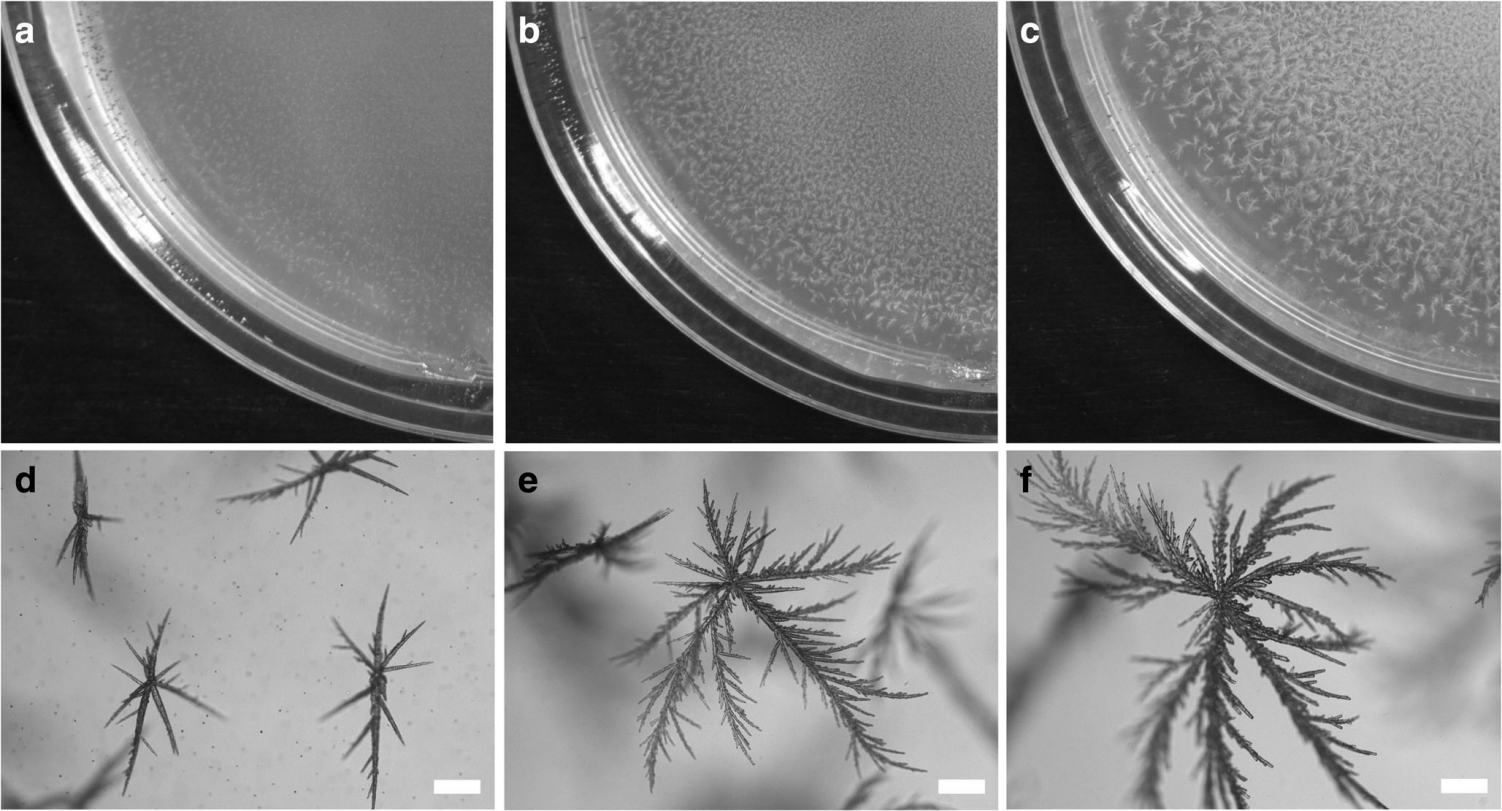
Formation of crystals in solid Czapek-Dox medium after 2 weeks incubation of *A. niger* at 25 °C in the dark. Plates were supplemented with **a**, **d** 1 mM, **b**, **e** 5 mM and **c**, **f** 10 mM LaCl_3_. (**d**–**f**, scale bars = 100 μm). Typical images are shown from several similar examinations

Obvious precipitation of white-coloured minerals was confirmed using light microscopy after 2 weeks of *A. niger* growth on MCD plates amended with 0, 1, 5 and 10 mM LaCl_3_ (Fig. 2a–c). It was observed that the quantity of precipitates increased with higher LaCl_3_ concentrations resulting in larger single crystals which appeared to be formed by continuous growth with branches ramifying in all directions with final sizes varying from approximately 300 to more than 1000 μm in length across three La concentrations (Fig. 2d–f).

### SEM and EDXA

**Fig. 3:**
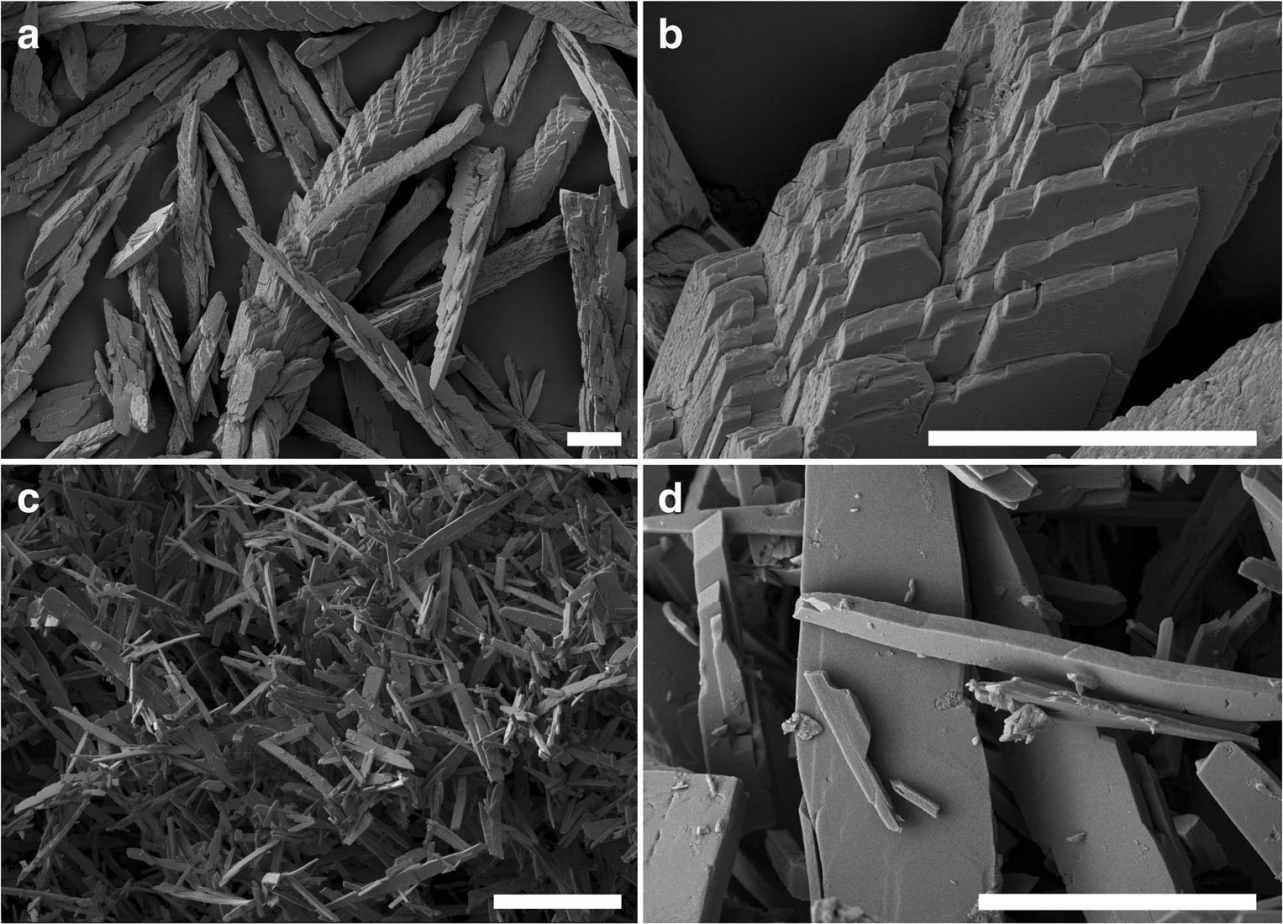
Scanning electron microscopy of **a**, **b** mycogenic crystals purified from 1 mM La-containing Czapek-Dox medium after 14-day incubation at 25 °C in the dark with *A. niger* and **c**, **d** crystals from chemical reaction of 25 mM oxalic acid and 1 mM lanthanum chloride. (**a**–**c**, scale bars = 50 μm; **d**, scale bar = 5 μm). Typical images are shown from several similar examinations

**Fig. 4:**
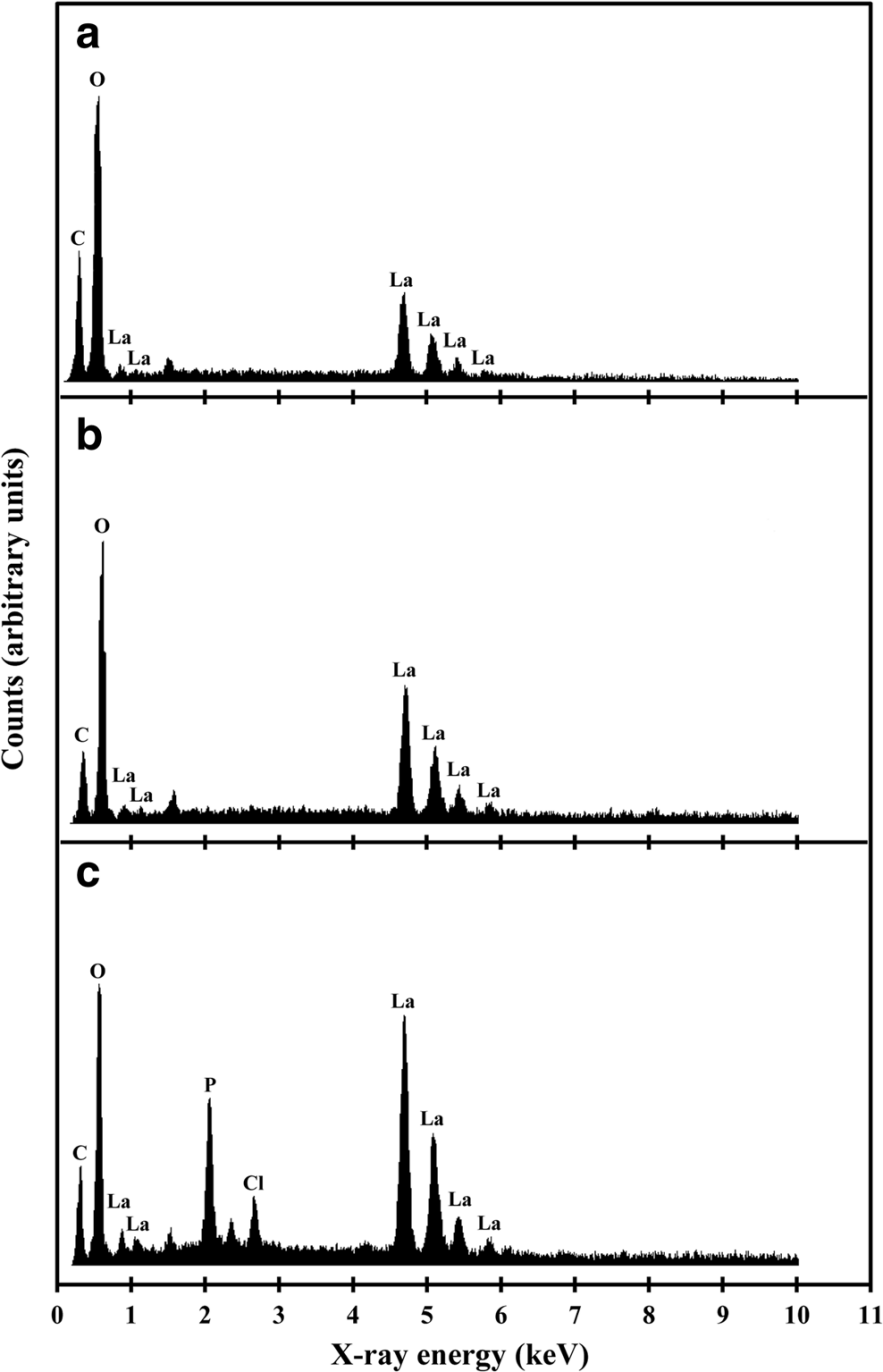
Energy-dispersive X-ray analysis of (**a**) mycogenic crystals formed in solid Czapek-Dox medium containing 1 mM LaCl_3_ after incubation with *A. niger* for 14 days at 25 °C in the dark; EDXA of crystals precipitated by reactions of (**b**) 1 mM and (**c**) 40 mM LaCl_3_ with biomass-free culture supernatant of NO_3_^−^-containing AP1 medium that was incubated with *A. niger* for 7 days at 25 °C in the dark. Typical spectra are shown from several similar determinations

Mycogenic crystals from MCD plates containing 1 mM LaCl_3_ were recovered after 14-day fungal growth and subjected to SEM. After the homogenization and washing processes, the large single crystal structures were disrupted into dendritic fragments measuring approximately 500 × 50 μm, and showing a layered texture (Fig. 3a, b). In contrast, crystals precipitated by an abiotic chemical reaction using pure oxalic acid and LaCl_3_ (Fig. 3c, d) consisted of only smooth crystal structures that did not show a layered pattern and were smaller (40 × 10 μm) than the mycogenic preparations. EDXA identified the main elements in the mycogenic crystals produced in solid (Fig. 4a) and liquid (Fig. 4b) media as C, O and La. The EDX spectra showed distinguishable peaks which matched well with the characteristic pattern of lanthanum at 0.833 and 4.650 keV. However, a phosphorus peak at 2.013 keV was evident in some samples produced from liquid reactions at high La concentrations (Fig. 4c).

### XRD analysis

**Fig. 5:**
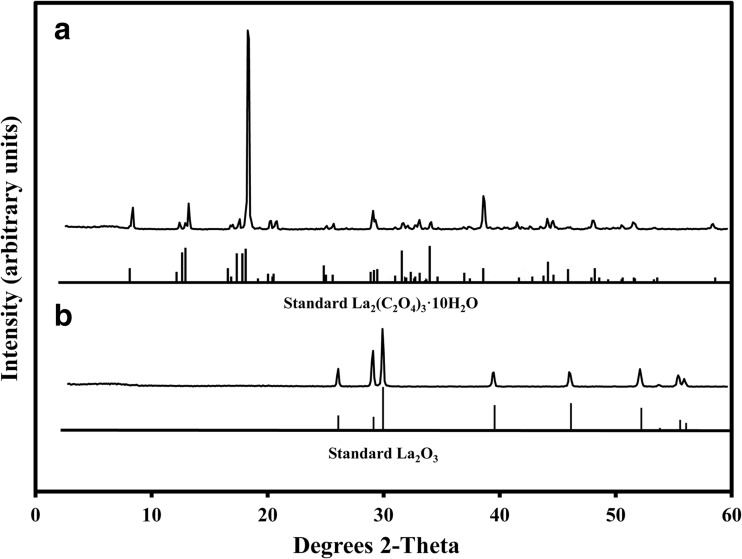
X-ray diffraction of (**a**) mycogenic crystals obtained from solid Czapek-Dox medium containing 1 mM LaCl_3_ and incubated with *A. niger* for 14 days at 25 °C in the dark; (**b**) XRD of the above-mentioned sample after thermogravimetric treatment at 1000 °C until constant weight. The standard patterns shown below the XRD patterns are (**a**) lanthanum oxalate decahydrate (JCPDS card no. 20-549) and (**b**) lanthanum oxide (JCPDS card no. 05-602). Typical patterns are shown from several similar determinations

The XRD pattern for the biogenic samples showed a well-formed crystalline structure with the peaks matching well with the standard pattern for La_2_(C_2_O_4_)_3_·10H_2_O (JCPDS card no. 20-549) in the database (Fig. 5a). Therefore, the mycogenic crystals were conclusively identified as lanthanum oxalate decahydrate.

### Thermal decomposition analysis

**Fig. 6:**
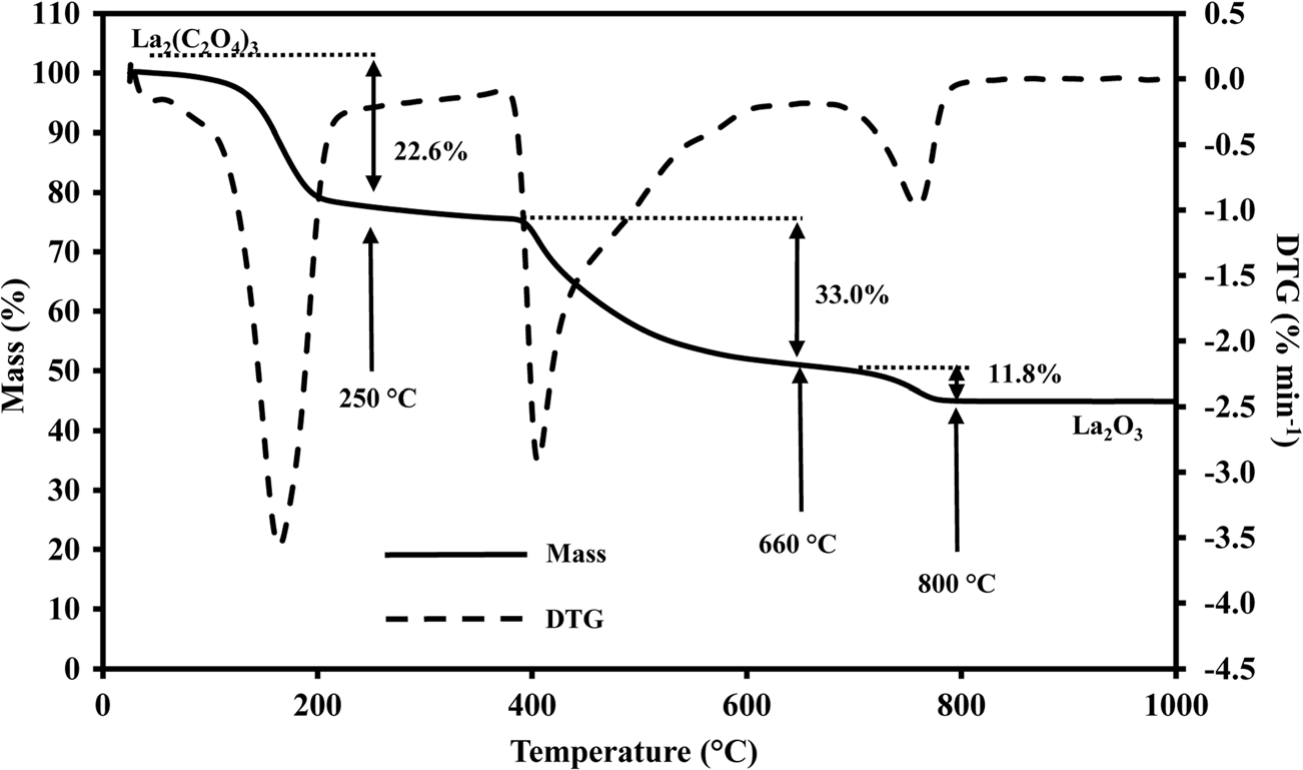
Thermogravimetric analysis of mycogenic crystals obtained from solid Czapek-Dox medium amended with 1 mM LaCl_3_ and incubated with *A. niger* for 14 days at 25 °C in the dark. DTG denotes differential thermal gravimetry, which is expressed as percentage of weight loss per minute. A typical pattern is shown from several similar determinations, all of which gave similar results

Thermogravimetric experiments recorded a stepwise thermal decomposition course for the mycogenic crystals, which was identical with that of abiotic controls. The decomposition process consisted of three stages commencing with an initial 22.6% weight loss from 45 to 250 °C, followed by 33.0% from 400 to 600 °C and 11.8% from 710 to 800 °C until the weight remained constant (Fig. 6). The total mass loss amounted to 54.6% at above 800 °C. The XRD pattern of the sample after complete thermal decomposition showed a high match with the standard pattern for La_2_O_3_ (JCPDS card no. 22-369) (Fig. 5b).

### Biomass-free culture supernatant experiments

**Fig. 7:**
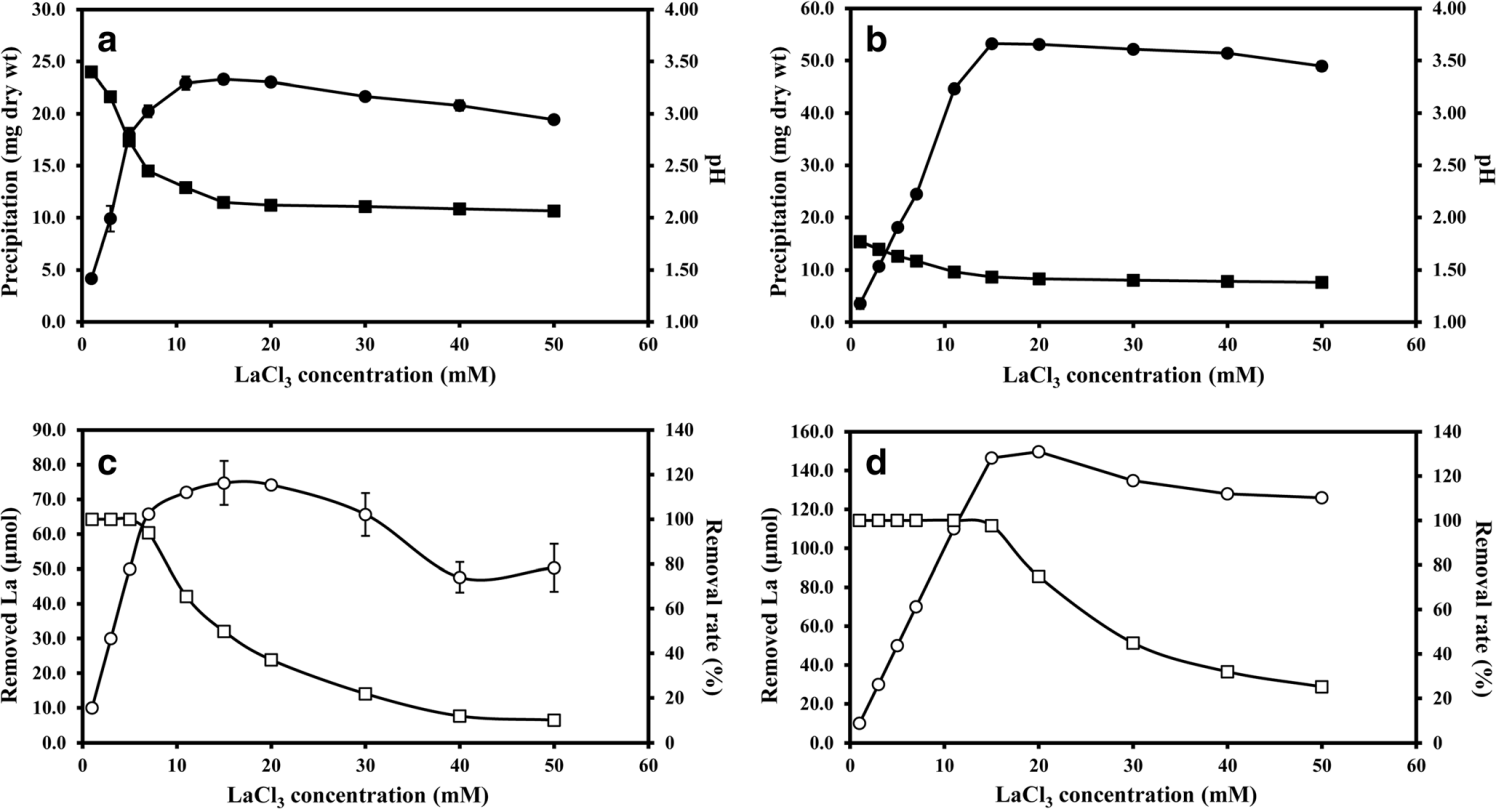
**a** Precipitation yield and **c** La removal rates for reactions of 1 ml LaCl_3_ and 9 ml biomass-free supernatant of NO_3_^−^-AP1 medium which was incubated with *A. niger* for 7 days at 25 °C in the dark. **b** Precipitation yield and **d** La removal rates for chemical reactions of 9 ml 25 mM oxalic acid with 1 ml LaCl_3_. The amount of La removed after liquid reactions (empty circle); dry wt of precipitate (filled circle); removal rate of La (empty square); pH after reaction (filled square). All data are given as means of at least three independent replicates. Standard deviation is represented by error bars which are not shown when smaller than the symbols

Reactions using mycogenic supernatants were performed using supernatants obtained from shaken liquid cultures of *A. niger* grown for 1 week in NO_3_^−^-containing AP1 medium. The pH of the supernatant had dropped to pH 3.41 ± 0.10 upon harvest. Large amounts of white precipitates were formed as a result of the 24 h reaction between LaCl_3_ and culture supernatants on the rotary shaker. At lower La concentrations, the precipitates were obviously granular and quickly settled at the tube bottom while at higher concentrations, they appeared to be smaller and formed a suspension. The production yield of precipitates from the 10-ml reaction system increased from 4.2 mg dry wt at 1 mM La to 23.3 mg dry wt at 15 mM La, which was the maximum yield observed. At concentrations higher than 15 mM La, the yield was slightly reduced to 23 mg at 20 mM La, 21.7 mg at 30 mM La, 20.8 mg at 40 mM La and 19.4 mg at 50 mM La. The pH, which was negatively correlated with increasing concentration of La, decreased from pH 3.40 at 1 mM La to pH 2.07 at 50 mM La. However, the decrease in pH was most prominent within the range 1 to 15 mM La and tended to remain unchanged at concentrations above 15 mM La (Fig. 7a). The percent removal of La was 100% with 1, 3 and 5 mM LaCl_3_ in the reaction system but was reduced at higher LaCl_3_ concentrations: 94.0% at 7 mM La, 65.5% at 11 mM La, 49.8% at 15 mM La, 37.1% at 20 mM La, 21.9% at 30 mM La, 11.9% at 40 mM La and 10.1% at 50 mM La. The maximum amount of La (74.8 μmol) was removed at 15 mM La (Fig. 7c). The kinetics of La removal showed a high similarity with those of precipitation yield.

Control experiments were carried out using 25 mM oxalic acid solution reacted with solutions of LaCl_3_. It was found that the precipitation yield in the control group also showed an increase with rising La concentrations until 15 mM La where the maximum yield of 53.3 mg dry wt was reached. A similar trend of pH change was found for the control which declined from pH 1.77 at 1 mM La to pH 1.43 at 15 mM La (Fig. 7b). The amount of removed La, which reached 146.5 mg dry wt, was only slightly lower than the maximum of 149.7 mg dry wt occurring at 20 mM and showed almost a linear relationship to the lanthanum concentration over the range 1 to 15 mM La. The removal rate was maintained at 100% until 11 mM La and slightly dropped to 97.7% at 15 mM La before a marked decline at 20 mM La and above (Fig. 7d).

**Fig. 8:**
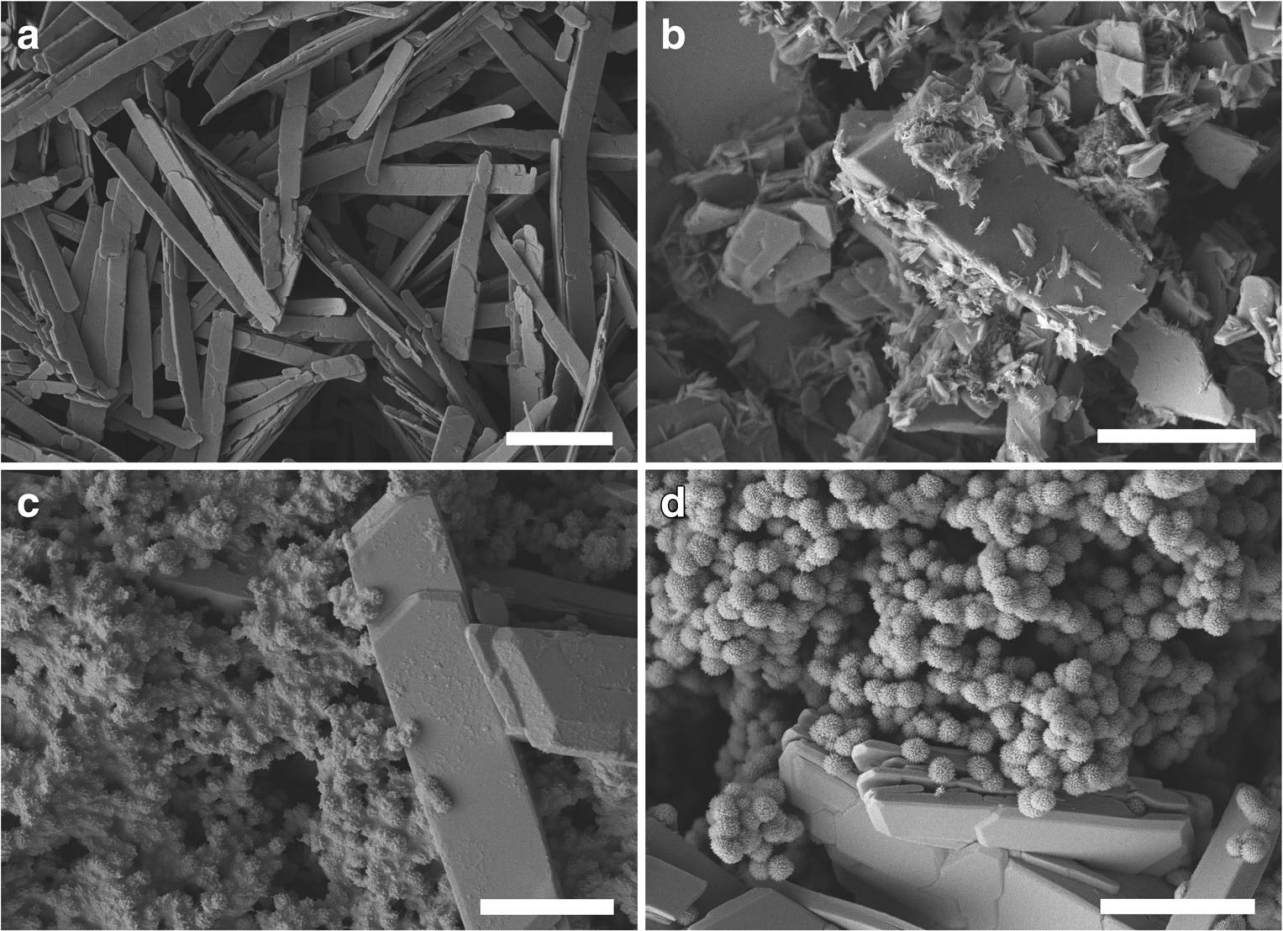
Scanning electron microscopy of crystal samples from reactions of **a** 1, **b** 7, **c** 30, and **d** 50 mM LaCl_3_ with biomass-free culture supernatant of NO_3_^−^-AP1 medium grown with *A. niger* for 7 days at 25 °C in the dark. (**a**–**d**, scale bars = 5 μm). Typical images are shown from several similar examinations

**Fig. 9:**
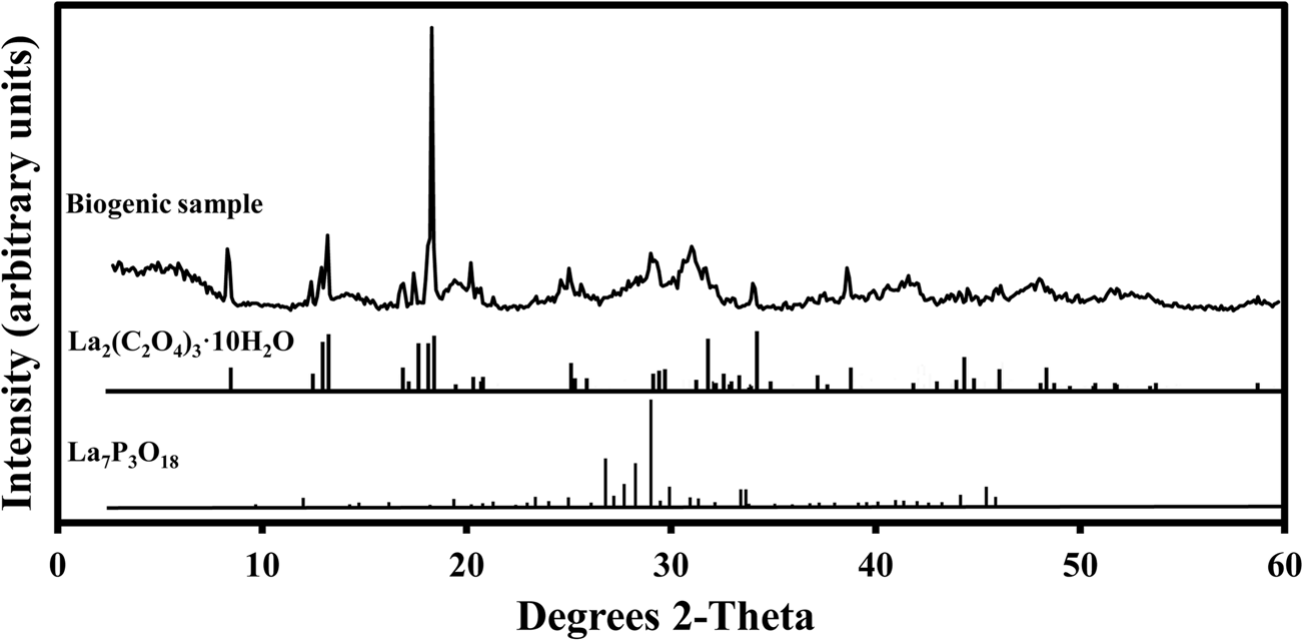
X-ray diffraction of crystals from reaction of 20 mM LaCl_3_ with biomass-free culture supernatant of NO_3_^−^-AP1 medium after incubation with *A. niger* for 7 days at 25 °C in the dark. Standard patterns of La_2_(C_2_O_4_)_3_·10H_2_O and La_7_P_3_O_18_ are also shown. A typical result is shown from one of several similar determinations, all of which gave similar results

SEM showed that at low La concentration (1 mM La), the crystals were of a relatively flat structure with smooth surfaces and measuring approximately 1 μm in width and 10 μm in length (Fig. 8a) and bore a close resemblance with those precipitated in the abiotic chemical reactions (Fig. 3d). At a medium La concentration (7 mM La), the crystals were larger in size and their surfaces were associated with small fragments of irregular shape (Fig. 8b). Aggregates of spherical minerals appeared, and the amount of tabular-shaped single crystals decreased at 20 mM La and above (Fig. 8c, d). All these spherical structures were of the same size measuring less than 1 μm in diameter. The mineral samples produced at both lower and higher La concentrations were subjected to further examination using EDXA and XRD. EDXA of samples obtained at higher La levels showed the presence of phosphorus, which was further confirmed by XRD showing well-matched patterns for both La_2_(C_2_O_4_)_3_·10H_2_O and the P-containing mineral La_7_P_3_O_8_ (Fig. 9).

### Geochemical modelling

**Fig. 10:**
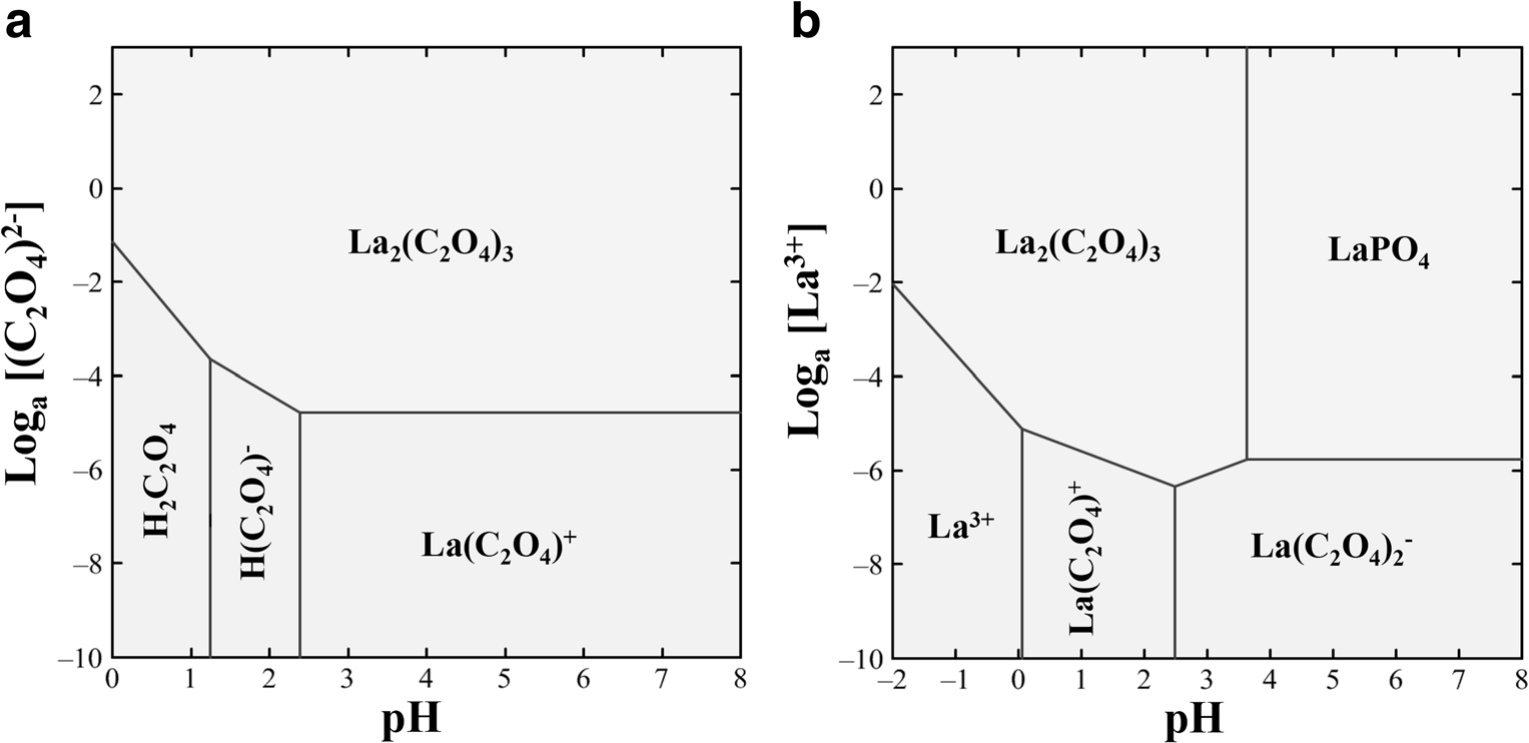
**a** Speciation diagram of pH versus log [(C_2_O_4_)^2−^] in the presence of 1 mM LaCl_3_ and **b** pH vs log [La^3+^] in the presence of 25 mM oxalic acid. All simulated reactions were set at 25 °C under 1.013 bars atmosphere pressure, and the activities of all chemicals contained in the media were calculated using Geochemist’s Workbench in accordance with their actual concentrations

The geochemical model for the solid medium experiments was constructed with the assumption that the system contained 1 mM LaCl_3_ in order to simulate the formation of lanthanum oxalate by the secretion of mycogenic oxalic acid. The model for the liquid culture reactions was created to simulate the precipitation of the biominerals in the presence of 25 mM oxalic acid, a realistic value to be released by *A. niger* (Ceci et al. 2015b). The results showed there was a difference in mineral phase speciation in both solid and liquid media. In solid medium, where lanthanum oxalate was the only solid phase in the modelled system, La_2_(C_2_O_4_)_3_ could be formed over the range 0 < pH < 2.39 if the concentration of (C_2_O_4_)^2−^ was above 10^–1.15^ M and at pH > 2.39 when the (C_2_O_4_)^2−^ concentration was above 10^-4.78^ M (Fig. 10a). In the liquid reaction systems, in addition to La_2_(C_2_O_4_)_3_, LaPO_4_ could be precipitated when the pH was above pH 3.63 and the La^3+^ concentration no less than 10^–5.77^ M (Fig. 10b).

## Discussion

Despite the chemical properties and characteristics of lanthanum and its compounds being well-studied in a chemical context, its interactions with microorganisms have received scant attention. Our work, for the first time, has demonstrated the formation of insoluble biogenic lanthanum oxalate by fungi and has therefore offered an insight into possible future biotechnological applications for the biorecovery of lanthanides and other rare earths and metals (Liang and Gadd 2017). Growth of *A. niger* in solid medium was only slightly affected by even high concentrations (> 10 mM) of lanthanum and the formation of large amounts of crystals was achieved, demonstrating the high efficiency of the process. This work has therefore laid a foundation for the biorecovery of REE using mycogenic oxalic acid. Crystals of lanthanum oxalate precipitated under chemical conditions usually carry 10.2 molecules of water and have a well-formed monoclinic crystal habit with clinopinacoid (0,1,0) (where the crystal plane is parallel to the vertical and the inclined lateral axes), orthopinacoid (1,0,0) (with crystal planes parallel to the orthodiagonal and vertical axes) and clinodome (0,1,1) (a dome in which the planes are parallel to the inclined axis) structures (Gilphin and McCrone 1952), which is in agreement with the morphological features of our samples produced at high La concentrations (Fig. 8c, d). The morphology of abiotic REE oxalates can also vary according to the methods and conditions applied to the precipitation reaction. Flower-like hierarchical microparticles, which showed similarity to those obtained in our work, were formed when La_2_(C_2_O_4_)_3_·10H_2_O was precipitated at room temperature in the presence of sodium citrate, and nanotubes were produced using a mixed solvent of water and ethanol in a 1:1 ratio (Zhang et al. 2014).

Thermogravimetric analysis is a useful tool to investigate the thermal properties of crystals as well as their structure and characterizes the thermal decomposition of biominerals (Balboul et al. 2002). Chlorides of REE are highly soluble in water and can be precipitated as oxalates, which may be further converted into oxides by ignition at 800 °C (Kolthoff and Elmquist 1931). The biogenic lanthanum oxalate crystals produced in the work underwent three stages of decomposition resulting in a total mass change of 54.6%, which is consistent with theoretical data (54.8%) based on calculations of the stepwise decomposition course as follows (Balboul et al. 2002) with lanthanum oxide (La_2_O_3_) being the final product:

1 $$ {\mathrm{La}}_2{\left({\mathrm{C}}_2{\mathrm{O}}_4\right)}_3\cdotp 10{\mathrm{H}}_2\mathrm{O}\to {\mathrm{La}}_2{\left({\mathrm{C}}_2{\mathrm{O}}_4\right)}_3\cdotp 4{\mathrm{H}}_2\mathrm{O}+6{\mathrm{H}}_2\mathrm{O}\kern0.5em {E}_a=65.47\ \mathrm{kJ}\ {\mathrm{mol}}^{-1} $$

2 $$ {\mathrm{La}}_2{\left({\mathrm{C}}_2{\mathrm{O}}_4\right)}_3\cdotp 4{\mathrm{H}}_2\mathrm{O}\to {\mathrm{La}}_2{\left({\mathrm{C}}_2{\mathrm{O}}_4\right)}_3\cdotp 2{\mathrm{H}}_2\mathrm{O}+2{\mathrm{H}}_2\mathrm{O}\kern0.5em {E}_a=106.90\ \mathrm{kJ}\ {\mathrm{mol}}^{-1} $$

3 $$ {\mathrm{La}}_2{\left({\mathrm{C}}_2{\mathrm{O}}_4\right)}_3\cdotp 2{\mathrm{H}}_2\mathrm{O}\to {\mathrm{La}}_2{\left({\mathrm{C}}_2{\mathrm{O}}_4\right)}_3+2{\mathrm{H}}_2\mathrm{O}\kern0.5em {E}_a=120.90\ \mathrm{kJ}\ {\mathrm{mol}}^{-1} $$

4 $$ {\mathrm{La}}_2{\left({\mathrm{C}}_2{\mathrm{O}}_4\right)}_3\to {\mathrm{La}}_2{\mathrm{O}}_2{\mathrm{C}\mathrm{O}}_3+3\mathrm{CO}\uparrow +2{\mathrm{C}\mathrm{O}}_2\uparrow \kern0.5em {E}_a=177.68\ \mathrm{kJ}\ {\mathrm{mol}}^{-1} $$

5 $$ {\mathrm{La}}_2{\mathrm{O}}_2{\mathrm{CO}}_3\to {\mathrm{La}}_2{\mathrm{O}}_3+{\mathrm{CO}}_2\uparrow \kern0.5em {E}_a=156.4\ \mathrm{kJ}\ {\mathrm{mol}}^{-1} $$

where *E*_*a*_ represents the energy of activation.

All five stages of thermal decomposition can generally be fitted to a kinetic function (Zhan et al. 2012):

6 $$ G\left(\alpha \right)={\left[1-{\left(1+\alpha \right)}^{1/3}\right]}^2 $$

where *α* is the extent of thermal conversion dependent on *E*_*a*_ and defined by the following equation:

7 $$ \alpha =\left({m}_0-m\right)/\left({m}_0-{m}_{\mathrm{f}}\right) $$

where *m* is the mass of the sample at a given time; *m*_0_ and *m*_f_ refer to the mass at the beginning and the end of the thermal decomposition.

The temperature range and decomposition course generally corresponded with a previous study where La_2_(C_2_O_4_)_3_·10H_2_O was heated to 900 °C and a weight loss of 58.1% was recorded (Zhan et al. 2012). However, a step of 4.9% mass loss occurred at 226 °C, which was caused by the transformation of hexahydate to dihydrate, was not prominent here and a total loss of 22.6% upon reaching 250 °C was recorded. This work has therefore not only demonstrated a means to recover La as lanthanum oxalate but also that mycogenic lanthanum oxalate can serve as a precursor for the preparation of La_2_O_3_ which has significant catalytic properties and is of use in several industrial fields including biodiesel production, manufacture of optical glass, phosphate removal and antimicrobial substances (Johnson et al. 2005; Liu et al. 2017; Fang et al. 2018; Salinas et al. 2018).

As regards geomicrobial transformations of minerals, fungal-produced oxalic acid can play a role in the solubilization of several minerals including calcite and dolomite (Gadd 2017). Minerals containing, e.g. Ca, Cd, Co, Cu, Mg, Mn, Sr, Zn, Ni and Pb can be transformed into oxalates by interaction with oxalate-secreting fungi (Gadd 2007; Sullivan et al. 2012; Gadd et al. 2014). Many preceding studies involving the transformation of minerals by oxalic acid-producing fungi have focused on the bioprecipitation of calcium oxalate as whewellite and weddellite (Punja and Jenkins 1984; Gadd et al. 2014; Gadd 2017). One of our preliminary experiments using La-containing malt extract agar as a growth medium for *A. niger* also developed pyramid-shaped crystals with typical morphological characteristics of calcium oxalate amid lanthanum oxalate crystals (data not shown). Oxalic acid could be useful in the final steps of metallurgical processes to recovery REE from leachates or other solutions (Sinha et al. 2016). Chi et al. (2004) applied oxalic acid to precipitate total rare earth elements, including Ce and La, from a leachate from bastnasite processing, and the produced oxalates were transformed into oxides after being roasted at 900 °C for 2 h. A similar process was also reported in another study where oxalic acid was utilized to recover lanthanides from REE-bearing phosphate rocks after being treated with strong acid (Aly and Mohammed 1999). Moreover, an appropriate amount of (NH_4_)_2_C_2_O_4_ was successfully used to separate lanthanum from a leachate consisting of a mixture of elements extracted from spent Ni-Mn batteries (Fernandes et al. 2013). Our XRD results showed uniformity for all biogenic and control samples from both agar plates and liquid media reactions at 1 mM La, confirming that the biominerals were well-formed crystalline structures of high purity as indicated by the sharp characteristic peaks matching impeccably with standard patterns for lanthanum oxalate in the database and a flat noise base.

In solid media experiments, only lanthanum oxalate crystals occurred even at high La concentrations. However, at higher La concentrations in the biomass-free liquid culture supernatant experiments, phosphorus was found in high amounts, associated with micro-sized spheres, indicating that P-containing compounds were also precipitated. This coincided well with the results of pH changes shown in the mass balance analysis at pH 3.40 and 1 mM La and with the geochemical modelling which showed lanthanum oxalate speciation when below pH 3.63. The plateau occurring above 15 mM La, as shown in the mass balance analysis, indicated that oxalic acid was depleted by excessive amounts of La^3+^, and any remaining La could participate in the precipitation of phosphates in the liquid media. It was reported that lanthanum phosphate, usually in the form of micro- or nano-sized particles and having a number of different mineral phases, could be produced by mixing La(NO_3_)_3_ and H_3_PO_4_ in a continuous precipitation reactor (Kawase et al. 2007). This could also account for the phosphates obtained at high La concentrations in our work. It is well known that fungi can release P from inorganic and organic sources which can therefore interact with other metals such as cadmium, cobalt, copper, lead, zinc and uranium, leading to the precipitation of secondary metal phosphates as microscale or nanoscale structures (Rhee et al. 2012, 2014; Gadd et al. 2014; Liang et al. 2015, 2016). In the present study, phosphates precipitated in the supernatant reaction system probably arose from chemical moieties in the growth medium according to the geochemical modelling where LaPO_4_ is formed if the system is pH > ~ 3.5 in the presence of excessive amounts of La^3+^ (Fig. 10b). Therefore, the precipitation of P-containing biominerals could be caused by the presence of phosphates in the supernatant: by adjusting the pH and the volume of the lanthanum solution, the formation of lanthanum phosphates could be avoided.

In conclusion, the fungal transformation of lanthanum chloride into La_2_(C_2_O_4_)_3_·10H_2_O, which can be subsequently converted into lanthanum oxide, is achievable by bioprecipitation in both La-containing solid media and biomass-free culture supernatants. Our study suggests a new biotechnological aspect for the biorecovery of rare earth elements.
